# Resilience of electricity grids against transmission line overloads under wind power injection at different nodes

**DOI:** 10.1038/s41598-017-11465-w

**Published:** 2017-09-14

**Authors:** Christoph Schiel, Pedro G. Lind, Philipp Maass

**Affiliations:** Fachbereich Physik, Universität Osnabrück, Barbarastrasse 7, 49076 Osnabrück, Germany

## Abstract

A steadily increasing fraction of renewable energy sources for electricity production requires a better understanding of how stochastic power generation affects the stability of electricity grids. Here, we assess the resilience of an IEEE test grid against single transmission line overloads under wind power injection based on the dc power flow equations and a quasi-static grid response to wind fluctuations. Thereby we focus on the mutual influence of wind power generation at different nodes. We find that overload probabilities vary strongly between different pairs of nodes and become highly affected by spatial correlations of wind fluctuations. An unexpected behaviour is uncovered: for a large number of node pairs, increasing wind power injection at one node can increase the power threshold at the other node with respect to line overloads in the grid. We find that this seemingly paradoxical behaviour is related to the topological distance of the overloaded line from the shortest path connecting the wind nodes. In the considered test grid, it occurs for all node pairs, where the overloaded line belongs to the shortest path.

## Introduction

With the constantly rising fraction of renewable energy sources in electricity production, it becomes an increasingly challenging task to make electricity grids most efficient and reliable. In particular, the embedding of renewable power is one major problem when planning and upgrading power grids in what concerns the size, location and distribution of renewable power plants^1^. To tackle this problem, a combination of methods developed in the fields of nonlinear dynamics, network theory and stochastic modelling provides a promising approach^2–5^. Moreover, new control designs are required^6^. Achievements have been made in optimal embedding forecast, using machine learning methods^7^, and for providing optimal grid structures in terms of good connection conditions^8^. Since one power grid can cover several countries, optimal solutions raise challenges on how to coordinate efforts between different countries, belonging to the European grid^9^.

Deriving embedding solutions is crucial for cost efficient design and stable functioning of the power grid^10^. A key element for stability of power grids is the adjustment of the generated power to the consumed power and power losses. In the absence of renewable energy sources, this adjustment needs to be ensured with respect to stochastic variations in the demands and failures of technology. Various control mechanisms have been implemented successfully in the past to maintain frequency and voltage stability, and reserve capacities were allocated to establish a resilience against failures of generators, transmission lines or other components of the grid^11^. Given the stochastic nature of wind and solar power, the stability against fluctuations needs to be considered also from the production side. Fluctuations in renewable power occur on time scales much smaller than the fluctuations in the power demand^12, 13^ and they pose new challenges for the control and optimisation of power grids. Their intermittent nature occurs on different spatial and time scales^13–15^ and is reflected in properties of wind turbines such as power and fatigue loads^16, 17^. These features raise questions on how control mechanisms need to be modified to guarantee the resilience and proper functioning of electricity grids in the future. One option to cope with this problem is to avoid a direct feed-in of renewable power into the grid, but to store the renewable energy before injecting it into the grid in a controlled way. However, this option can be expensive and limited by the available amount of storage facilities. A direct feed-in of renewable power, on the other hand, must be supplemented by power from conventional generators to keep control over the balancing of generated and consumed power. Therefore, in view of plans to substitute a considerable fraction of the total generated power by renewable energy^18^, it becomes important to study favourable embedding strategies of renewable energy sources into existing power grids^19, 20^.

**Figure 1:**
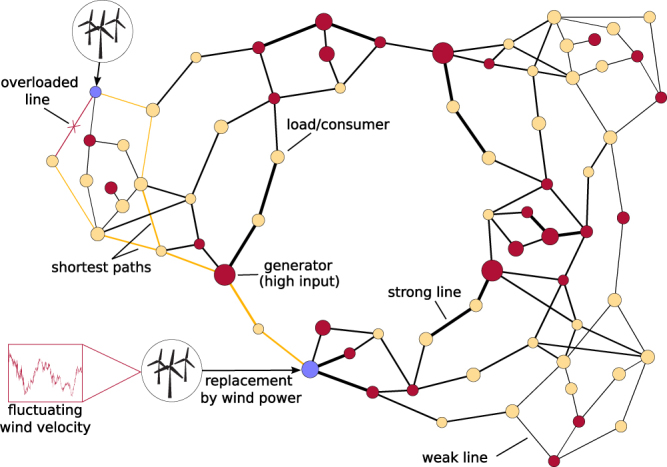
Illustration of the methodology for estimating line overload probabilities under wind power injection in the IEEE RTS-96 test grid. The IEEE RTS-96 test grid consists of 30 generator nodes (red circles, dark grey), 41 load nodes (yellow circles, bright grey) and 108 transmission lines. Wind power is injected by replacing conventional generators as indicated (blue/grey circles). If the injected wind power becomes too strong, line overload occurs, as indicated by the red (dark) overloaded line. The radii of the circles is drawn proportional to the total power generated/consumed at the respective nodes (as listed in the IEEE data set, i.e. before replacement of conventional generators), and the thickness of the transmission lines is proportional to their maximum capacity. The two paths of connecting lines coloured in yellow (bright grey) mark shortest paths between the two wind feeding nodes.

In this paper, we study how wind power feeding at different nodes of a power grid affects its stability against overloads of transmission lines. Such overloading can lead to overheating and line overload. Specifically we address the following question: If a given amount of conventionally generated power shall be replaced by wind power, where are the most favourable locations for wind farms if single line overloads shall be avoided? The methodology of our approach follows partly previous work^19^ and is illustrated in Fig. 1. Wind power is injected at the generator nodes of an IEEE test grid by replacing nodes of conventional generators. Thereby, heterogeneities in the power production and consumption, as well as in the transmission line properties are taken into account, and we embed the wind power in a topological environment typical for a generator node. For the distribution of the fluctuating wind power we choose a Weibull distribution. This is motivated by empirical results for wind velocities and the so-called “power curve”^21^, which describes the average relation between wind velocity and power. A quasi-static response of the grid state with respect to the wind fluctuations is considered and the balancing of generated and consumed power is ensured by scaling the power input from the remaining (non-substituted) conventional generators. For our analysis, the IEEE RTS-96 test grid^22^ is taken as an example.

After describing the methods more specifically, we first address the question how strongly the resilience against line overloads varies with the location of wind power injection, if exactly one conventional generator in the grid is replaced by a wind farm. It turns out that the grid resilience can be quite sensitive to the location of the injection node. For a wind farm with average power production of 200 MW, we find the overload probabilities to vary over more than two orders of magnitude. Nodes with highest overload probabilities have at most one generator node as neighbour and they have a comparatively low total capacity of their emanating transmission lines. We then study how simultaneous wind power input at different nodes affects the grid resilience against line overloads. Therefore, pairs of the conventional generators are replaced by wind farms with fluctuating power generation. We find that a higher wind power input at one of the injection nodes can increase the threshold power for line overload at the other node. This surprising behaviour is correlated with the distance of the overloaded line from the shortest path connecting the pair of wind nodes. Finally, we show that spatial correlations between wind power fluctuations at the two injection nodes need to be taken into account in order to identify the best pairs with lowest overload probabilities. We conclude the paper with a summary of the key results, their impact for applications and an outlook for further investigations.

## Methods

### Feasibility regions

The power flow in the test grid is treated based on a linearised version of the ac power flow equations, which is an often applied procedure in the electric engineering literature^23^. In this approximation, three simplifications are made: (i) the resistances of the transmission lines [*jk*] between nodes *j* and *k* are neglected in comparison to their reactances, implying that their properties can be fully described by susceptances ${b}_{jk}={b}_{kj}$, with ${b}_{lm}=0$ for all pairs of nodes *l* and *m* that are not connected, (ii) the moduli $|{V}_{j}|$ of the complex voltages ${V}_{j}=|{V}_{j}|{e}^{i{\theta }_{j}}$ at the nodes $j=\mathrm{1,}\ldots ,N$ are considered to be constant and here set to one, and (iii) the voltage phase differences between nodes connected by a transmission line are assumed to be much smaller than one, $({\theta }_{j}-{\theta }_{k})\ll 1$. Setting $|{V}_{j}|=1$ in reference units, the (real) powers *P*_*j*_ ejected from nodes *j* are then connected to the voltage phase angles by the dc power flow equations

1 $${P}_{j}=\sum _{k\mathrm{=1}}^{N}{b}_{jk}({\theta }_{k}-{\theta }_{j}),\quad j=\mathrm{1,}\ldots ,N.$$

In the IEEE test grid data, powers ${P}_{j}^{({\rm{g}})} > 0$ of generators and ${P}_{j}^{({\rm{d}})} < 0$ of consumers (demands) are listed for a typical situation, and yield the net power ${P}_{j}={{P}_{j}}^{({\rm{g}})}+{P}_{j}^{({\rm{d}})}$ at each node *j*. Due to the lossless transmission in the dc power flow, there is a balancing of total generated and consumed power ${\sum }_{j}{P}_{j}=0$, which follows also from the symmetry of the *b*_*jk*_ and Eq. (1). By choosing one voltage phase angle as the reference, e. g., *θ*_*N*_ = 0, Eq. (1) are solved to express the remaining *N* − 1 phase angles *θ*_*j*_ in terms of the *N* − 1 independent *P*_*j*_ (from power balancing, ${P}_{N}=-{\sum }_{j\mathrm{=1}}^{N-1}{P}_{j}$). Knowing the phase angles *θ*_*j*_, we can calculate the power flows ${P}_{jk}\equiv {b}_{jk}({\theta }_{k}-{\theta }_{j})$ along each transmission line [*jk*]. These flows are limited by a maximal capacity ${P}_{[jk]}^{{\rm{\max }}}$ for each transmission line,

2 $$|{P}_{jk}|=|{b}_{jk}({\theta }_{k}-{\theta }_{j})| < {P}_{[jk]}^{{\rm{\max }}}.$$

If $|{P}_{jk}|\ge {P}_{[jk]}^{{\rm{\max }}}$, the transmission line [*jk*] is considered to be overloaded.

In studying the stability of the grid under additional injection of wind energy, we follow previous work^19^ and assume that the fluctuations of wind power occur on time scales short compared to that of load fluctuations and long compared to time scales needed for power adjustment of conventional generators. Accordingly, the consumed powers ${P}_{j}^{({\rm{d}})}$ are taken to be constant. For including wind energy, we replace $n$ of the ${P}_{j}^{({\rm{g}})}$ by powers ${g}_{l} > 0$ from wind farms, $l=\mathrm{1,}\ldots ,n$, and rescale the remaining ones with a common factor to ensure ${\sum }_{j}{P}_{j}=0$. This rescaling can be viewed as a simple means to account for droop control or regulation response^19^. As for the values of the ${P}_{[jk]}^{{\rm{\max }}}$ in Eq. (2), we use the short-time emergency ratings of the *IEEE Reliability Test System 1996* (IEEE RTS-96). This test grid was developed for comparative and benchmark studies^22^ and provides 15 tables of data. In Table 1 we list the entries in the IEEE RTS-96 data set, from which the relevant information for this work was extracted.

**Table 1:** Information extracted from the IEEE RTS-96 test grid.

Information	Source location
Power demand ${{P}_{j}}^{({\rm{d}})}$	Table 1, column 4
Power generation ${{P}_{j}}^{({\rm{g}})}$	Table 7, column 4
Lines $[jk]$	Table 12, columns 2 and 3
Reactances ${x}_{jk}={b}_{jk}^{-1}$	Table 12, column 9
Rating ${P}_{[jk]}^{{\rm{\max }}}$	Table 12, column 13

Given a replacement, the phase angles from the solutions of Eq. (1) become linear functions of the wind powers,

3 $${\theta }_{j}={\alpha }_{j}+\sum _{l\mathrm{=1}}^{n}{\beta }_{jl}\,{g}_{l},\quad j=\mathrm{1,}\ldots ,N-\mathrm{1,}$$

with coefficients *α*_*j*_, *β*_*jl*_ depending on the ${P}_{j}^{({\rm{d}})}$, the non-substituted ${P}_{j}^{({\rm{g}})}$ and the set of susceptances of the transmission lines. Inserting these solutions into the conditions (2) yields, for each transmission line, feasibility regions in an $n$-dimensional Cartesian space ${{\mathbb{R}}}_{+}^{n}$ spanned by the wind powers. For the line [*jk*], this region is bounded by the two limiting planes $({\alpha }_{k}-{\alpha }_{j})+{\sum }_{l}^{n}({\beta }_{kl}-{\beta }_{jl}){g}_{l}=\pm {P}_{[jk]}^{max}/|{b}_{jk}|$. Considering the set of all such limiting planes, there is a subset, which, together with the coordinate planes, confines a convex $n$-polytope around the origin of the wind power space, which is not intersected by any of the limiting planes. This polytope constitutes the feasibility region with respect to a line overload anywhere in the grid. If the $n$ wind powers $({g}_{1},\ldots ,{g}_{n})$ lie inside this region, no overload occurs. Otherwise, at least one transmission line overloads.

In this work, we consider either one (n = 1) or two (n = 2) wind farms. Then the polytope becomes a line (n = 1) or polygon (n = 1). The line is given by a threshold value *g*_*i*_^(*c*)^ for each possible wind node *i*, and the polygons are denoted as *P*_*ij*_ for the possible pairs (*i*, *j*) of injection nodes.

### Statistics of wind power fluctuations and line overload probabilities

To capture the statistics of wind power fluctuations is a difficult task that requires a good model for the transformation of wind speed into wind power (as performed by wind mills) and a description of the wind velocity statistics in the turbulent flow of the atmosphere, which shows long-ranged temporal and spatial correlations. There is continuing progress in the modelling of these issues^13, 16, 24–26^ but this progress has not yet matured to a state of established standard models. Here we base our description on empirical findings for the distribution of wind velocities and on the known average relation between wind speed and power characterised by the power curve^21, 27^. As for the spatial correlations between wind powers at different nodes, we consider the two extremes of completely uncorrelated and completely correlated fluctuations. This allows us to gain insight into the importance of such correlations for the probability of line overloads.

To be specific, we take the Weibull distribution

4 $${\mathscr{W}}(x;k,\lambda )=\frac{k}{\lambda }\,{(\frac{x}{\lambda })}^{k-1}\exp [-{(\frac{x}{\lambda })}^{k}]\,,$$

for the probability distribution function (PDF) ${\rho }_{v}(v)$ of wind velocities with a shape parameter ${k}_{v}\simeq 2$, as reported in the literature^28, 29^, ${\rho }_{v}(v)={\mathscr{W}}(v;2,{\lambda }_{v})$. In contrast to the shape parameter, the scale parameter ${\lambda }_{v}$ depends significantly on the location of the wind farm, because locations with comparable ${k}_{v}\simeq 2$ and stronger mean wind speed must have larger ${\lambda }_{v}$. This variation, however, is not relevant in our modelling approach, because we consider a situation, where a given mean amount of wind power ${\bar{g}}^{({\rm{t}}{\rm{o}}{\rm{t}})}$ shall be injected into the grid. Different ${\lambda }_{v}$ then amount to different wind farm sizes.

According to the power-curve, the power $g$ of a wind farm is on average proportional to the cube of the wind speed $v$ over the most relevant range of velocities^27^, where wind turbines operate. Taking $g\propto {v}^{3}$, the PDF $\rho (g)$ of wind powers becomes a Weibull distribution as well with shape parameter $k={k}_{v}/3\simeq 2/3$, i. e. $\rho (g;\lambda )={\mathscr{W}}(g;2/3,\lambda )$.

If one single wind farm is included into the grid, the scale parameter $\lambda ={\lambda }_{i}$ will be fixed for injection node $i$ by the condition

5 $${\bar{g}}_{i}^{({\rm{t}}{\rm{o}}{\rm{t}})}={\int }_{0}^{{g}_{i}^{({\rm{c}})}}{\rm{d}}g\rho (g)g={\int }_{0}^{{g}_{i}^{({\rm{c}})}}{\rm{d}}g{\mathscr{W}}(g;{\textstyle \tfrac{2}{3}},{\lambda }_{i})g=\frac{3}{2}\,{\lambda }_{i}[\frac{\sqrt{\pi }}{2}-\frac{2}{3}\,{\rm{\Gamma }}(\frac{5}{2},{(\frac{{g}_{i}^{({\rm{c}})}}{{\lambda }_{i}})}^{2/3})],$$

where $\Gamma (\cdot ,\cdot )$ is the incomplete Gamma function^30^,

6 $$\Gamma (a,x)={\int }_{0}^{x}{y}^{a-1}{e}^{-y}dy.$$

This equation is solved numerically for ${\lambda }_{i}$. Knowing ${\lambda }_{i}$, we obtain the line overload probability

7 $${{\rm{\Pi }}}_{i}=1-{\int }_{0}^{{g}_{i}^{({\rm{c}})}}{\rm{d}}g\rho (g)=1-{\int }_{0}^{{g}_{i}^{({\rm{c}})}}{\rm{d}}g{\mathscr{W}}(g;{\textstyle \tfrac{2}{3}},{\lambda }_{i})=\exp [-{(\frac{{g}_{i}^{({\rm{c}})}}{{\lambda }_{i}})}^{2/3}]$$

for wind power feeding at node *i*.

In the case of two wind farms, we need to specify the joint probability density ${\rho }_{2}({g}_{1},{g}_{2})$ for the powers *g*_1_ and *g*_2_ at the two wind farms. For a given pair of wind nodes (*i*, *j*), this gives the line overload probability

8 $${\Pi }_{ij}=1-{\int }_{0}^{\infty }{\rm{d}}{g}_{1}{\int }_{0}^{\infty }{\rm{d}}{g}_{2}\,{I}_{ij}({g}_{1},{g}_{2})\,{\rho }_{2}({g}_{1},{g}_{2})\,,$$

where ${I}_{ij}({g}_{1},{g}_{2})$ is the indicator function of the feasibility region ${{\mathscr{P}}}_{ij}$, i. e., ${I}_{ij}({g}_{1},{g}_{2})=1$ for $({g}_{1},{g}_{2})\in {{\mathscr{P}}}_{ij}$ and zero otherwise. For uncorrelated wind velocity fluctuations at the two wind farm locations we have

9 $${\rho }_{2}({g}_{1},{g}_{2})={\mathscr{W}}({g}_{1};\tfrac{2}{3},{\lambda }_{1}){\mathscr{W}}({g}_{2};\tfrac{2}{3},{\lambda }_{2}).$$

For completely correlated wind velocity fluctuations, i. e. equal wind speeds at the two wind farm locations, we can write ${g}_{1}={\lambda }_{1}\,{g}_{0}$ and ${g}_{2}={\lambda }_{2}\,{g}_{0}$, where ${g}_{0}$ is a reference power, as, e. g., given by the transformation of speed into power by one wind mill (of the same type in the two farms). Different scale parameters ${\lambda }_{1}\ne {\lambda }_{2}$ take into account that the farms at the two locations can have a different size. Hence, we have ${g}_{2}{\lambda }_{1}={g}_{1}{\lambda }_{2}$ as a constraint, which implies that the conditional probability of power ${g}_{2}$ for given power ${g}_{1}$ is ${\rho }_{\mathrm{1|1}}({g}_{2}|{g}_{1})=\delta ({g}_{2}-{g}_{1}{\lambda }_{2}/{\lambda }_{1})$. The joint probability density then becomes

10 $${\rho }_{2}({g}_{1},{g}_{2})=\rho ({g}_{1})\,{\rho }_{1|1}({g}_{2}|{g}_{1})={\mathscr{W}}({g}_{1};{\textstyle \tfrac{2}{3}},{\lambda }_{1})\,\delta ({g}_{2}-{g}_{1}{\lambda }_{2}/{\lambda }_{1})\,.$$

In both the uncorrelated and correlated case, the two scale parameters $({\lambda }_{i},{\lambda }_{j})$ are fixed by minimising the overload probability ${\Pi }_{ij}$ in Eq. (8) under the constraint

11 $${\bar{g}}_{ij}^{({\rm{t}}{\rm{o}}{\rm{t}})}={\int }_{0}^{{\rm{\infty }}}{\rm{d}}{g}_{1}{\int }_{0}^{{\rm{\infty }}}{\rm{d}}{g}_{2}\,{I}_{ij}({g}_{1},{g}_{2})\,{\rho }_{2}({g}_{1},{g}_{2})({g}_{1}+{g}_{2})\,.$$

The minimisation is performed by using the MATLAB Interior Point algorithm^31^ and corresponds to an optimisation of the wind farm sizes, if wind power with total mean amount ${\bar{g}}_{ij}^{({\rm{t}}{\rm{o}}{\rm{t}})}$ shall be injected at the nodes $i$ and $j$. Hence, when comparing line overload probabilities for different pairs of wind feeding nodes, this optimisation is always implicitly assumed. We finally note that the ratio ${\lambda }_{j}/{\lambda }_{i}$ in the correlated case is given by the slope of the longest line between the origin and one of the other corner points of ${{\mathscr{P}}}_{ij}$.

## Results

### Grid resilience under wind power injection at a single node

**Figure 2:**
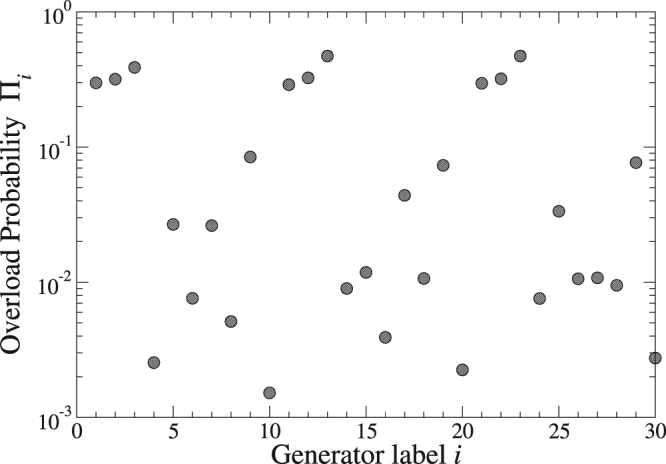
Variation of line overload probabilities for wind power injection at one node. For an average power production of 200 MW by the wind farm, the overload probabilities vary by more than two orders of magnitude for different wind feeding nodes.

As described in the Methods, a critical power ${g}_{i}^{({\rm{c}})}$ defines the feasibility region for wind power injection at a single node $i$: For $g\in [0,{g}_{i}^{({\rm{c}})}]$ no overload occurs in the grid, while for $g > {g}_{i}^{({\rm{c}})}$ at least one transmission line overloads. Figure 2 shows the corresponding overload probabilities ${\Pi }_{i}$ calculated from Eq. (7), where the ${\lambda }_{i}$ were determined from Eq. (5) for a wind farm size with an average power production ${\bar{g}}_{ij}^{({\rm{t}}{\rm{o}}{\rm{t}})}=200\,{\rm{M}}{\rm{W}}$ for all pairs. This corresponds to a power production of a mid-sized wind farm and also to the typical power production of the individual conventional generators in the test grid. The overload probabilities ${\Pi }_{i}$ vary by more than two orders of magnitude dependent on the selected injection node, showing that the protection against line overload can be an important factor for optimising wind power integration into existing grids. We found two structural features of the nodes to be particularly relevant for the magnitude of the associated overload probabilities. First, a fluctuation of high wind power should be more easily compensated by the grid, if it can be distributed among many strong lines in the immediate neighbourhood of the injection node. Analysing this correlation, we found the logarithms of the overload probabilities to linearly correlate with the capacity-weighted node degree with a Pearson correlation coefficient of −0.922. Secondly, conventional generator nodes in the neighbourhood of the wind node can help to stabilise the grid against line overload, because their regulated response implies a decrease of their power supply in the environment of the wind node upon increase of injected wind power. The most vulnerable nodes with high ${\Pi }_{i}$ in Fig. 2 have at most one conventional generator as neighbour and a small capacity-weighted node degree. The capacity-weighted node degrees for these nodes are even very similar in values, leading to seemingly repeated motifs of the corresponding ${\Pi }_{ij}$ in Fig. 2.

### Grid resilience under wind power injection at two nodes

**Figure 3:**
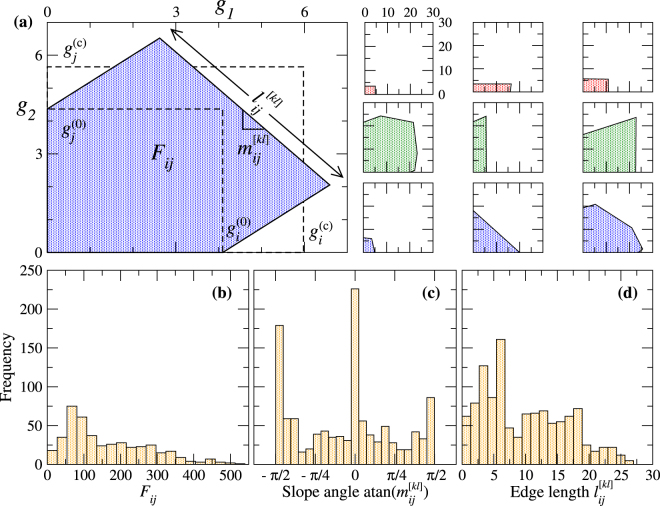
Shapes of feasibility regions and their statistics under wind power injection at two nodes. (**a**) Representative examples of feasibility regions (polygons) ${{\mathscr{P}}}_{ij}$. The polygon on the left side is drawn in large scale for illustration and corresponds to wind power injections ${g}_{1}$ and ${g}_{2}$ at nodes $i=1$ and $j=2$. Each edge of this polygon not lying on the ${g}_{1}$- or ${g}_{2}$-axis corresponds to a transmission line $[kl]$ that overloads if the wind power production $({g}_{1},{g}_{2})$ passes this edge. The edge associated with transmission line $[kl]$ has length ${l}_{ij}^{[kl]}$ and its slope ${m}_{ij}^{[kl]}$ characterises the coupling between the two wind nodes with respect to an overload of the line $[kl]$. The two rectangles marked by the dashed lines are drawn for comparison (see text). The nine small polygons arranged in an array on the right side are examples of feasibility regions for node pairs $(i,j)$ with topological distances ${L}_{ij}\mathrm{=3}$ (lower row), 5 (middle row), and 7 (upper row). Powers are given in of units of 100 MW. (**b**)-(**d**): Histograms of (**b**) the polygon areas ${F}_{ij}$, (**c**) the slope angles ${\rm{atan}}({m}_{ij}^{[kl]})$, and (**d**) the edge lengths ${l}_{ij}^{[kl]}$.

For wind power injection $({g}_{1},{g}_{2})$ at two nodes $i$ and $j$, the feasibility regions are polygons ${{\mathscr{P}}}_{ij}$: If $({g}_{1},{g}_{2})\in {{\mathscr{P}}}_{ij}$ the grid is stable, otherwise a line overload occurs. In our model setup, $435$ different polygons exist for the IEEE RTS-96 test grid, corresponding to the number of different pairings of distinct generator nodes. Representative examples of these polygons are shown in Fig. 3(a). The large polygon is the feasibility region for the wind feeding nodes $i=1$ and $j=2$, and the nine smaller polygons in the array refer to node pairs with different topological distances ${L}_{ij}$ (three polygon examples for three fixed distances ${L}_{ij}$): In the lower row, ${L}_{ij}=3$, in the middle row, ${L}_{ij}=5$, and in the upper row, ${L}_{ij}=7$.

Let us first consider the areas ${F}_{ij}$ of the polygons, which give a rough measure for overload probabilities in the case of uncorrelated powers injected at the two wind farms [cf. Eqs. (8) and (9)]. The examples in Fig. 3(a) show that these areas can be quite different for given topological distance ${L}_{ij}$. The histogram of all ${F}_{ij}$ in Fig. 3(c) displays a broad unimodal distribution with the mode at about ${(800{\textstyle \text{MW}})}^{2}$. The large spread of areas implies strong variations of overload probabilities for uncorrelated wind power feeding (see below) and hence reflects the relevance of proper node selection already seen in the case of single-node injection.

Interesting information on correlation properties of the two wind nodes with respect to line overload is provided by the shapes of the polygons. If wind power injection at one node would not affect the critical power injection for line overload at the other node, the polygons become simple rectangles. For comparison, two of such virtual rectangles, $[0,{g}_{i}^{\mathrm{(0)}}]\times [0,{g}_{j}^{\mathrm{(0)}}]$ and $[0,{g}_{i}^{({\rm{c}})}]\times [0,{g}_{j}^{({\rm{c}})}]$ are indicated by the dashed lines for the polygon drawn in large scale in Fig. 3(a). Here, the value ${g}_{i}^{\mathrm{(0)}}$ is the critical value for power injection at node $i$, if no power is generated at node $j$, and ${g}_{j}^{\mathrm{(0)}}$ has the analogous meaning. The value ${g}_{i}^{\mathrm{(0)}}$ differs from the critical value ${g}_{i}^{({\rm{c}})}$ for single node injection, because the latter refers to a situation, when there is a nonzero conventionally generated power at node $j$. With increasing topological distances ${L}_{ij}$ between the wind nodes, correlations decrease and the polygons tend to exhibit a more rectangular shape, see Fig. 3(a).

More detailed information on the correlations can be inferred by analysing the edges of the polygons. The two edges along the coordinate axes ${g}_{1}=0$ and ${g}_{2}=0$ are due to the constraints ${g}_{2}\ge 0$ and ${g}_{1}\ge 0$, respectively, while the other edges are associated with overloads of transmission lines. If the wind powers ${g}_{1}$ and ${g}_{2}$ at the injection nodes $i$ and $j$ are driven out of the feasibility region due to high wind velocities at one or both injection nodes, one edge of the polygon ${{\mathscr{P}}}_{ij}$ is passed, and the line $[kl]$ associated with this edge becomes overloaded. The slope ${m}_{ij}^{[kl]}$ of this edge, or the corresponding slope angle ${\rm{atan}}({m}_{ij}^{[kl]})\in [-\pi \mathrm{/2},\pi \mathrm{/2}[$, is a measure for the wind node coupling with respect to the overload of the line $[kl]$. For slope angles near zero (nearly horizontal edge) and $(\pm \pi \mathrm{/2)}$ (nearly vertical edge), a change of power at one injection node has almost no effect on the threshold power for line overload at the other node (as it is strictly the case for the edges in the rectangles). A negative slope means that an increase of wind power at one of the injection nodes decreases the threshold power at the other injection node for the overload of line $[kl]$. We refer to this expectable behaviour as negative node coupling. Interestingly, we also see in Fig. 3(a) that an increase of wind power at one injection node can rise the threshold power for line overload at the other injection node, i.e. it makes the grid more stable. This seemingly paradoxical behaviour occurs for positive slope angles and we then say that the wind nodes exhibit a positive coupling with respect to the overload of line $[kl]$. In a way, this resembles another peculiar behaviour known as Braess paradox^32^, where the addition of a transmission line, or an increase of its capacity, makes the grid less stable or weakens the flow.

Histograms of slope angles ${\rm{atan}}({m}_{ij}^{[kl]})$ and edge lengths ${l}_{ij}^{[kl]}$ from all edges of all polygons are displayed in Fig. 3(c) and (d), respectively. While the histogram of edge length shows a wide spread of ${l}_{ij}^{[kl]}$ in the range of 100-2000 MW without characteristic signatures, the histogram of slope angles exhibits pronounced peaks at the bins close to the angles zero (horizontal edges) and $(\pm \pi \mathrm{/2)}$ (vertical edges). Minima appear close to the angles $(\pm \pi \mathrm{/4)}$, where the edges have maximum negative or positive coupling.

**Figure 4:**
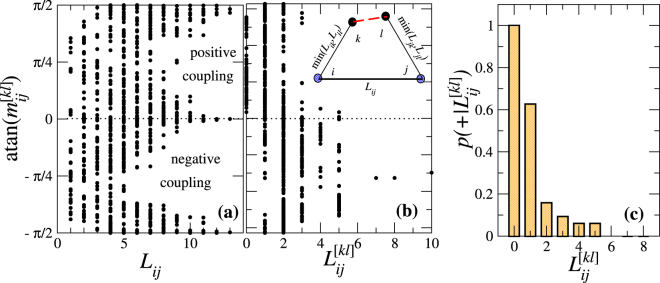
Character of node coupling in dependence of node distance and distance of overloaded line from shortest path. Plots of the slope angles ${\rm{atan}}({m}_{ij}^{[kl]})$ versus (**a**) the topological distance ${L}_{ij}$ between the wind feeding nodes $i$ and $j$, and (**b**) the distance ${L}_{ij}^{[kl]}$ of the overloaded line $[kl]$ from the shortest path connecting the wind nodes, according to the measure introduced in Eq. (12), which is illustrated in the inset. (**c**) The probability $p(+|{L}_{ij}^{[kl]})$ of finding a positive ($+$) node coupling under the condition of given ${L}_{ij}^{[kl]}$ is shown in dependence of ${L}_{ij}^{[kl]}$. For overloaded lines on the shortest path (${L}_{ij}^{[kl]}\mathrm{=0}$), the injection nodes $i$ and $j$ are always positively coupled.

Closer inspection of the distribution of the slope angles in dependence of the distances ${L}_{ij}$ between the wind nodes reveals that strong node couplings with ${\rm{atan}}({m}_{ij}^{[kl]})\simeq \pm \pi \mathrm{/4}$ occur only for sufficiently small ${L}_{ij}$. This is shown in Fig. 4(a), where all angles ${\rm{atan}}({m}_{ij}^{[kl]})$ are plotted against the distances ${L}_{ij}$. With increasing ${L}_{ij}$, the angle distribution separates into two peaks around zero and $(\pm \pi \mathrm{/2)}$ (in a repeated scheme, the figure may be viewed as periodically continued along the slope angle axis). Overall, the couplings become small with increasing distance ${L}_{ij}$.

The reason for the possibility to obtain positive couplings is that flows are directional and accordingly power injection from the wind nodes can compensate each other along a transmission line. If the overloaded line is on the shortest path connecting the two farms, one would therefore expect a compensating effect to occur with higher probability. More generally, we expect the positive couplings to be the more likely the closer the overload line lies on the shortest path. To quantify this feature, we introduce the following measure for the distance of the link $[kl]$ to the shortest path connecting nodes $i$ and $j$:

12 $${L}_{ij}^{[kl]}\equiv {\textstyle \text{min}}({L}_{ik},{L}_{il})+{\textstyle \text{min}}({L}_{jk},{L}_{jl})+1-{L}_{ij}\,.$$

This measure corresponds to the excess length when subtracting the distance ${L}_{ij}$ from the length of the path connecting nodes $i$ and $j$ via link $[kl]$, see the inset of Fig. 4(b). In particular, if the line $[kl]$ belongs to the shortest path, one has ${L}_{ij}^{[kl]}\mathrm{=0}$. In Fig. 4(b) the slope angles ${\rm{atan}}({m}_{ij}^{[kl]})$ are plotted against the distances ${L}_{ij}^{[kl]}$. Interestingly, positive node couplings become the more likely the smaller ${L}_{ij}^{[kl]}$. If the overloaded line $[kl]$ belongs to the shortest path connecting the wind nodes (${L}_{ij}^{[kl]}=0$), positive node couplings are even found for all these lines. As shown in Fig. 4(c), the probability $p(+|{L}_{ij}^{[kl]})$ of finding positive node coupling under the condition of given ${L}_{ij}^{[kl]}$ rapidly decreases from one for ${L}_{ij}^{[kl]}=0$ to zero for ${L}_{ij}^{[kl]}\ge 6$.

Mathematically, the slope ${m}_{ij}^{[kl]}$ for the overload of line $[kl]$ under wind power injection at nodes $i$ and $j$ follows from the solution of the dc power flow equations after the replacement of conventional generator nodes. If, for wind power feeding at nodes $i$ and $j$, Eq. (3) is written in the form (with the superscript $(i,j)$ labelling the dependence on the injection node numbers)

13 $${\theta }_{m}^{(i,j)}={\alpha }_{m}^{(i,j)}+{\beta }_{m1}^{(i,j)}{g}_{1}+{\beta }_{m2}^{(i,j)}{g}_{2}\,,\quad m=\mathrm{1,}\ldots ,N-\mathrm{1,}$$

we obtain

14 $${m}_{ij}^{[kl]}=-\frac{{\beta }_{k1}^{(i,j)}-{\beta }_{l1}^{(i,j)}}{{\beta }_{k2}^{(i,j)}-{\beta }_{l2}^{(i,j)}}.$$

The coefficients ${\beta }_{kn}^{(i,j)}$, $n=1,2$, thus mediate the interplay of the power flows coming from the wind nodes. Intuitively, one can understand the enhanced likelihood of positive node coupling for small ${L}_{ij}^{[kl]}$, or at least for ${L}_{ij}^{[kl]}=0$, by the following argument: Flow emanating from a generator node $i$ can be viewed as spreading in the “topological space” of the grid. Consequently, one should expect (with a high probability) that an increase in the generated power at node $i$ leads to an increase of flow along the link $[kl]$ in the direction $k$ to $l$, if node $k$ is closer to node $i$ than node $l$, *i.e*. ${L}_{il}-{L}_{ik}=1$, and to a decrease of flow in the same direction, if ${L}_{il}-{L}_{ik}=-1$. The analogue holds true for the power generated at node $j$. Therefore, if $({L}_{il}-{L}_{ik})({L}_{jl}-{L}_{jk})=-1$, power increments at the two nodes $i$ and $j$ likely are giving rise to mutually compensating flows through link $[kl]$. On the shortest path between nodes $i$ and $j$, all links $[kl]$ satisfy $({L}_{il}-{L}_{ik})({L}_{jl}-{L}_{jk})=-1$, and accordingly $p(+|{L}_{ij}^{[kl]}=0)=1$.

**Figure 5:**
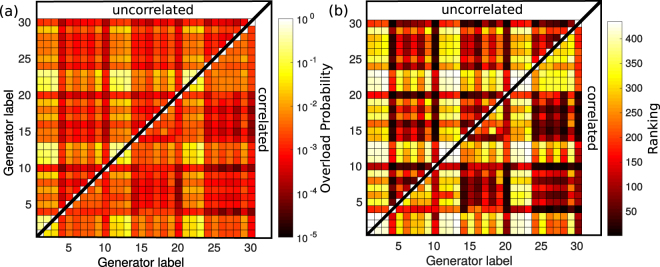
Effect of wind power correlations on line overload probabilities. (**a**) Outage probability and (**b**) ranking of the grid resilience against line overload for all possible pairings of wind feeding nodes and the same average total wind power production of ${\bar{g}}_{ij}^{({\rm{t}}{\rm{o}}{\rm{t}})}=200\,{\textstyle \text{MW}}$ as in Fig. 2 (now generated by two wind farms). In each of the two arrays, the upper/lower triangle shows the results for uncorrelated/correlated wind powers at the two farm locations.

Finally, we analyse the impact of spatial correlations between the wind powers at the injection nodes on the line overload probabilities ${\Pi }_{ij}$. We choose the same average total wind power production of ${\bar{g}}_{ij}^{({\rm{t}}{\rm{o}}{\rm{t}})}=200\,{\textstyle \text{MW}}$ as considered in Fig. 2 for the single node injection. This average total power is generated now by two wind farms, where the individual farm sizes are optimised to yield the lowest possible overload probability ${\Pi }_{ij}$ (see Methods). For comparing the uncorrelated with the correlated case, we present the overload probabilities in an array in Fig. 5(a), where for each possible pair $(i,j)$ of injection nodes, ${\Pi }_{ij}$ is plotted in a colour scale (see the scale bar in the figure). In the upper triangle of the array above its diagonal, the ${\Pi }_{ij}$ are given for the uncorrelated case, and in the lower triangle for the correlated case.

As a first result from Fig. 5(a) we find that the smallest ${\Pi }_{ij}$ are by about two orders of magnitude smaller than in Fig. 2 for single-node injection, which demonstrates the advantage of decentralised wind power generation for the grid stability (for same average total wind power feeding). The injection nodes 4, 10, 16, 20, and 30, yielding the lowest line overload probability under single node injection, cf. Figure 2, give also comparatively low overload probabilities when paired with another injection node, both in the uncorrelated and correlated case. Corresponding rows and columns of the $\Pi$ matrix in Fig. 5(a) remain in the red (dark grey) regime of overload probabilities in the range ${10}^{-5}-{10}^{-3}$ (with one exception for node number $i=20$ in the correlated case, which shows higher ${\Pi }_{ij}$ when paired with nodes $j=21$, 22, or 23). That pairs of nodes tend to give low ${\Pi }_{ij}$ if one of its nodes yields a low overload probability as a single wind power source, is a consequence of our optimisation of the wind farm sizes: If a “good node” $i$ (${\Pi }_{i}$ low) is paired with a “bad node” $j$ (${\Pi }_{j}$ high), ${\Pi }_{ij}$ can be avoided to become very high by increasing the farm size at node $i$.

These common features for the correlated and uncorrelated case, however, do not imply that the best pairs of injection nodes are the same for uncorrelated and correlated wind powers. The five pairs yielding lowest ${\Pi }_{ij}$ are listed in Table 2. While one of the nodes with numbers 4, 10, and 30 appears in all pairings for both the uncorrelated and correlated case, its “pairing node” is always different, i.e. none of the five best pairs in the uncorrelated case agrees with one of the five best pairs in the correlated case. This demonstrates the relevance of wind power correlations in the search for optimal wind feeding nodes. Pairs of the same rank in Table 2 have about 2-3 times lower overload probabilities for correlated wind powers. For completeness, we show in Fig. 5(b) the ranking of all node pairs with respect to grid resilience against line overload.

**Table 2:** Node pairs for wind power injection with the lowest line overload probabilities.

Uncorrelated	${\Pi }_{ij}\times {10}^{5}$	Correlated	${\Pi }_{ij}\times {10}^{5}$
4, 10	7.35	10, 28	4.35
4, 20	13.6	4, 28	5.31
10, 16	15.6	10, 26	5.97
10, 24	17.0	4, 26	6.82
20, 30	17.1	18, 30	8.27

The five most optimal pairs of nodes for wind power feeding yielding the lowest probabilities for line overload in the gird. Different optimal pairs are obtained for uncorrelated and correlated wind powers at the two wind farm locations.

## Discussion and Outlook

In this work we studied the line overload probabilities under fluctuating wind power injection at different nodes of an IEEE test grid in a model setup, where one or two conventional generators are replaced by wind feeding nodes with given total average power production. A quasi-static response of the power flow to wind fluctuations was assumed and we calculated this flow from the dc power flow equations under the constraint of total balance between generated and consumed power. This was achieved by a corresponding rescaling of the controllable generators. To describe the distribution of wind speeds, we used the Weibull distribution with a typical shape parameter $k=2$ reported in the literature. By resorting to the known average relation between wind speed and power, this was transformed into a Weibull distribution of wind power with shape parameter $k=\mathrm{2/3}$. For two injection nodes we considered the two limiting cases of uncorrelated and completely correlated wind powers at the wind farm locations to get insight into the role of large-scale spatial correlations of wind fluctuations. Scale parameters of the Weibull distributions were optimised by adjusting the wind farm sizes to yield lowest overload probabilities in the case of two-node injection (at given total wind power injection).

The main findings of our study can be summarised as follows: (i) The overload probabilities vary strongly with the location of injection nodes as a consequence of the heterogeneous grid properties, showing their importance for optimal integration of wind energy. A reduction of overload probabilities by about two orders of magnitude is seen if the same average amount of wind power is injected via two nodes rather than a single node. This gives an estimate of the benefit of decentralising wind energy integration for avoiding line overloads. (ii) An analysis of the structure of feasibility regions in the two-node injection case allows one to obtain valuable insight into couplings between injection nodes with respect to transmission line overloads, which are independent of the detailed wind statistics. In particular, many positive couplings exist, where an increase of wind power at one injection node increases the threshold power for line overload at the other node. It was found that node pairs with positive couplings need to be topologically close to each other and that overloaded lines for positively coupled injection nodes tend to lay along the shortest path between these nodes. The first feature can be understood from the decrease of coupling strength with injection node distance, and the second feature from the likelihood that flows emanating from the injection nodes mutually compensate each other along transmission lines close to the shortest path. (iii) Large-scale spatial correlations between wind fluctuations seem to be a relevant factor for optimal integration of wind power. We found them to change the best pairs of nodes yielding lowest overload probabilities, and for these pairs to reduce the risk of line overload by a factor of two to three.

Generally, it is an important goal to provide proper tools and measures for estimating risks of transmission line overloads under increasing integration of renewable energy sources into power grids, and to develop risk-minimising strategies for the design of new grid structures that are better suitable for transporting electric energy between a large number of small power sources. The methodology used in this study, where we followed previous work reported in ref. 19 is just one step towards these goals. An important issue to be clarified in future studies is how far the quasi-static approach can be considered to be valid. This question is intimately connected with the magnitude of various time scales, in particular those specifying the power response to a change in wind speed and the correlations of wind speed fluctuations. It needs to be checked also how far the primary stability control on the scale of seconds^11^ can be effectively accounted for by a simple power rescaling of controllable generators. We have started to investigate these problems by conducting dynamical studies based on swing equations for the IEEE RTS-96 test grid, i.e. the same test grid as used in this study. When succeeding to identify a time scale of the validity of the quasi-static approach it should become possible to connect the calculated values of the line overload probabilities to time intervals, where one can expect a line overload to occur. In the present study we have refrained to pursue any attempts in this direction and regarded the calculated overload probabilities solely as a relative measure for comparison of different wind power injections. Moreover, we have not considered the specific function that specifies the power curve of one wind turbine or one wind farm. It is known that this average function has a saturation level, above the so-called rated speed^21^, which is specific for each wind turbine and needs to be taken into account for calculating the costs associated with maintenance. For estimating overload probabilities with the proposed methodology, the saturation effect can be considered in the transformation of the velocity distribution (4) to the power distribution. However, a significant effect on the overload probabilities is only to be expected if the derivation from the Weibull distribution are pronounced in the feasibility regions.

Apart from these necessary checks, we believe that our concept of positive and negative node couplings with respect to flow through transmission lines can be a valuable general tool to qualify the mutual influencing of power injections. The concept can be generalised to more than two power injections ($n\mathrm{ > 2}$). In this case, the feasibility region for power injection at $n$ given nodes is an $n$-dimensional polytope with $(n-\mathrm{1)}$-dimensional hyperplanes confining it, and the set of coefficients ${\beta }_{rs}$, $r=\mathrm{1,}\ldots ,n$, $l=\mathrm{1,}\ldots ,N-1$, appearing in Eq. (3) define normal vectors to these planes, i. e. specify their orientations. For a transmission line $[kl]$, a pair $(i,j)$ of the $n$ injection nodes has a positive coupling, if $({\beta }_{ki}-{\beta }_{li})$ and $({\beta }_{kj}-{\beta }_{lj})$ have opposite signs, and negative coupling otherwise. One may ask then, for example, how strongly the character of node coupling changes with the addition of injection nodes and how far the combined effect of many nodes on the flow can be traced back to pairwise couplings.

The results of our work can help to reveal the influence brought by renewable energy sources on the electricity grid. For applications, it is also important to estimate costs and returns, and to develop optimisation strategies with respect to the expected gain. Such optimisations will depend also on specific objectives of an investor and hence should involve expertise from economists working in this field. To illustrate how the overload probabilities can enter corresponding optimisation problems, let us consider a simple approach, where we introduce two different kinds of costs, fixed costs, and maintenance costs that vary in time. The fixed costs ${c}_{i}^{({\rm{c}}{\rm{u}})}({\lambda }_{i})$ and ${c}_{j}^{({\rm{c}}{\rm{u}})}({\lambda }_{j})$ are due to construction or upgrading and depend on the parameters ${\lambda }_{i}$ and ${\lambda }_{j}$ controlling the farm sizes. The maintenance cost per time unit will also depend on the farm sizes, and we denote them as ${c}_{i}^{({\rm{m}})}({\lambda }_{i})$ and ${c}_{j}^{({\rm{m}})}({\lambda }_{j})$. The total costs when implementing renewable sources at nodes $i$ and $j$ can then be defined as

15 $${C}_{ij}^{({\rm{t}}{\rm{o}}{\rm{t}})}({\lambda }_{i},{\lambda }_{j})={c}_{i}^{({\rm{c}}{\rm{u}})}({\lambda }_{i})+{c}_{j}^{({\rm{c}}{\rm{u}})}({\lambda }_{j})+[{\kappa }_{ij}{{\rm{\Pi }}}_{ij}({\lambda }_{i},{\lambda }_{j})+{c}_{i}^{({\rm{m}})}({\lambda }_{i})+{c}_{j}^{({\rm{m}})}({\lambda }_{j})]\tau ,$$

where $\tau$ is the time horizon for the investment. The term ${\kappa }_{ij}{\Pi }_{ij}$ is the average rate of overloads, with ${\kappa }_{ij}$ a proportionality factor. This proportionality factor depends on the injection nodes due to the costs depending on the overload lines $[kl]$ forming the edges of the feasibility polygon, including an average over the respective lines $[kl]$. The overload probability ${\Pi }_{ij}$ is given by Eq. (8), where now we state explicitly the dependence on the farm sizes, ${g}_{ij}={\lambda }_{ij}\,{g}_{0}$. Complementary to the total amount of costs, one has the expected return, which can be considered to depend on the total power production per unit time

16 $${R}_{ij}({\lambda }_{i},{\lambda }_{j})=\tau {r}_{ij}{\bar{g}}_{ij}^{({\rm{t}}{\rm{o}}{\rm{t}})}({\lambda }_{i},{\lambda }_{j}).$$

Both the total cost and the return can be determined from the sizes ${\lambda }_{i}$ and ${\lambda }_{j}$ of the nodes where power is injected in the grid. Accordingly from these functions one can calculate the gain

17 $${{\rm{\Omega }}}_{ij}({\lambda }_{i},{\lambda }_{j})=\frac{{R}_{ij}({\lambda }_{i},{\lambda }_{j})}{{C}_{ij}^{({\rm{t}}{\rm{o}}{\rm{t}})}({\lambda }_{i},{\lambda }_{j})},$$

and optimize this with respect to the farm sizes. The best pair $(i,j)$ of nodes is the one with the largest ${{\rm{\Omega }}}_{ij}$. The full framework to account simultaneously for cost and overload probability could also be extended to incorporate an explicit time dependence of the maintenance costs as well as of the overload probability and the total power production.

### Data Availability

The data that support the findings of this study are available from reference 22 and also at https://www2.ee.washington.edu/research/pstca/rts/pg_tcarts.htm.
